# Canadian Expert Panel Recommendations for MRI Use in MS Diagnosis and Monitoring

**DOI:** 10.1017/cjn.2015.24

**Published:** 2015-05

**Authors:** Anthony Traboulsee, Laurent Létourneau-Guillon, Mark Steven Freedman, Paul W. O’Connor, Aditya Bharatha, Santanu Chakraborty, J. Marc Girard, Fabrizio Giuliani, John T. Lysack, James J. Marriott, Luanne M. Metz, Sarah A. Morrow, Jiwon Oh, Manas Sharma, Robert A. Vandorpe, Talia Alexandra Vertinsky, Vikram S. Wadhwa, Sarah von Riedemann, David K.B. Li

**Affiliations:** 1 Division of Neurology, UBC Hospital/University of British Columbia, Vancouver, British Columbia; 2 Université de Montréal Neuroradiologue/Neuroradiologist - Hôpital Notre-Dame, Centre Hospitalier de l'Université de Montréal, Montreal, Quebec, Canada; 3 Department of Neurology, The Ottawa Hospital/University of Ottawa and the Ottawa Hospital Research Institute, Ottawa, Ontario, Canada; 4 Multiple Sclerosis (MS) Clinic and Evoked Potentials Laboratory, Medical Advisory – MS Society of Canada, and Department of Medicine - University of Toronto, Toronto, Ontario; 5 Keenan Research Centre of the Li Ka Shing Knowledge Institute, St. Michael’s Hospital, Toronto, Ontario, Canada; 6 Division of Neuroradiology, Department of Radiology, The Ottawa Hospital/University of Ottawa, Ottawa, Ontario, Canada; 7 Centre Hospitalier de l'Université de Montréal/ Department of Neuroscience, Université de Montréal, Montréal, Québec, Canada; 8 Division of Neurology, 4D Kaye Edmonton Clinic, University of Alberta, Edmonton, Alberta, Canada; 9 Radiology, Clinical Neurosciences, and Surgery, Foothills Medical Centre/University of Calgary, Calgary, Alberta, Canada; 10 Department of Internal Medicine (Neurology), Health Sciences Centre/University of Manitoba, Winnipeg, Manitoba, Canada; 11 Department of Clinical Neurosciences, University of Calgary, Calgary, Alberta, Canada; 12 University of Western Ontario MS Clinic, London Health Sciences Centre/Western University, London, Ontario, Canada; 13 Faculty of Medicine (Neurology), St. Michael’s Hospital/University of Toronto, Toronto, Ontario, Canada; 14 Department of Medical Imaging, London Health Sciences Centre/Western University, London, Ontario, Canada; 15 Department of Diagnostic Radiology, QEII Health Sciences Centre/Dalhousie University, Halifax, Nova Scotia, Canada; 16 Department of Radiology, Vancouver General Hospital/University of British Columbia, Vancouver, British Columbia, Canada; 17 Department of Radiology, Health Sciences Centre/University of Manitoba, Winnipeg, Manitoba, Canada; 18 SCRIPT, Toronto, Ontario, Canada; 19 Department of Radiology/Division of Neurology, University of British Columbia, Vancouver, British Columbia, Canada

**Keywords:** Magnetic resonance imaging, multiple sclerosis, multiple sclerosis MRI, neuroimaging

## Abstract

***Background***: A definitive diagnosis of multiple sclerosis (MS), as distinct from a clinically isolated syndrome, requires one of two conditions: a second clinical attack or particular magnetic resonance imaging (MRI) findings as defined by the McDonald criteria. MRI is also important after a diagnosis is made as a means of monitoring subclinical disease activity. While a standardized protocol for diagnostic and follow-up MRI has been developed by the Consortium of Multiple Sclerosis Centres, acceptance and implementation in Canada have been suboptimal. ***Methods***: To improve diagnosis, monitoring, and management of a clinically isolated syndrome and MS, a Canadian expert panel created consensus recommendations about the appropriate application of the 2010 McDonald criteria in routine practice, strategies to improve adherence to the standardized Consortium of Multiple Sclerosis Centres MRI protocol, and methods for ensuring effective communication among health care practitioners, in particular referring physicians, neurologists, and radiologists. ***Results***: This article presents eight consensus statements developed by the expert panel, along with the rationale underlying the recommendations and commentaries on how to prioritize resource use within the Canadian healthcare system. ***Conclusions***: The expert panel calls on neurologists and radiologists in Canada to incorporate the McDonald criteria, the Consortium of Multiple Sclerosis Centres MRI protocol, and other guidance given in this consensus presentation into their practices. By improving communication and general awareness of best practices for MRI use in MS diagnosis and monitoring, we can improve patient care across Canada by providing timely diagnosis, informed management decisions, and better continuity of care.

## RÉSUMÉ

**Recommandations du Groupe consultatif canadien sur l’utilisation de l’IRM pour le diagnostic et la surveillance de la SP.** ***Contexte:*** Pour poser un diagnostic formel de sclérose en plaques (SP), par opposition à un syndrome isolé au point de vue clinique, une des deux conditions suivantes doit être remplie : une deuxième poussée clinique ou des constatations particulières à l’imagerie par résonance magnétique (IRM), selon les critères de McDonald. L’IRM est également importante après que le diagnostic ait été posé, comme moyen de surveiller l’activité subclinique de la maladie. Bien qu’un protocole standardisé pour le diagnostic et le suivi par l’IRM ait été développé par le Consortium of Multiple Sclerosis Centres, son acceptation et son application ont été sous-optimales au Canada. ***Méthode:*** Afin d’améliorer le diagnostic, la surveillance et la prise en charge d’un syndrome isolé au point de vue clinique et de la SP, un Groupe consultatif canadien d’experts a émis des recommandations consensus sur l’application appropriée des critères de McDonald (2010) en pratique clinique, des stratégies pour améliorer la fidélité au Consortium of Multiple Sclerosis Centres MRI protocol standardisé et des méthodes pour garantir la communication efficace entre les professionnels de la santé, particulièrement les médecins référents, les neurologues et les radiologistes. ***Résultats:*** Cet article présente huit énoncés de consensus développés par un groupe d’experts ainsi que le fondement de ces recommandations et des commentaires sur la façon de prioriser l’utilisation des ressources dans le cadre du système de santé canadien. ***Conclusions:*** Le groupe d’experts invite les neurologues et les radiologistes du Canada à incorporer dans leur pratique les critères de McDonald, le protocole d’IRM du Consortium of Multiple Sclerosis Centres ainsi que les autres directives émises dans le présent consensus. En améliorant la communication et en sensibilisant les professionnels de la santé au sujet de l’utilisation optimale de l’IRM pour le diagnostic et la surveillance de la SP, nous pouvons améliorer les soins aux patients à travers le Canada en établissant un diagnostic rapide, des décisions de traitement éclairées et une meilleure continuité dans les soins prodigués aux patients.

Multiple sclerosis (MS) is a chronic neurologic disease characterized by inflammatory activity and associated demyelinating damage (lesions or plaques). Lesions may occur in any part of the central nervous system, but are typically observed in the cerebellum, cerebrum, brainstem, basal ganglia, optic nerve, periventricular and juxtacortical white matter, and spinal cord.^1^ ^,^ ^2^ Neurological symptoms are diverse and may present either as isolated attacks (relapsing–remitting MS, RRMS) or as part of a steady progression.

MRI of the brain and/or spinal cord is an accepted standard of care, along with clinical evaluation, to establish a diagnosis of MS; it can also be used to monitor disease activity.^3^ Overall lesion number and disease burden can be visualized as hyperintense areas on T2-weighted and fluid-attenuated inversion recovery (FLAIR) scans, whereas T1-weighted scans can reveal “black holes” (T1-hypointense lesions), which generally indicate older, inactive lesions associated with greater permanent axonal damage. T1-weighted scans are sometimes performed with the contrast agent gadolinium to identify new lesions that represent areas of active inflammation with blood–brain barrier breakdown. The FLAIR pulse sequence is particularly useful in brain MRI because cerebrospinal fluid appears dark, making it easier to distinguish from bright-appearing lesions.^4^ FLAIR is also valuable for detection of some cortical and juxtacortical lesions.^5^

## Dissemination in Time and in Space

Because of the multifaceted nature of MS and its symptoms, differential diagnosis is complex. Over the years, various efforts have been made to standardize diagnostic criteria and ensure consistent definitions. The initial Schumacher criteria of 1965 included three key concepts that persist to this day—that for a patient’s condition to be identified as MS, there must be:

• Dissemination in space (DIS): Lesions in at least two different anatomical areas;
• Dissemination in time (DIT): In the case of the Schumacher criteria, at least two distinct clinical attacks separated by at least 30 days; and
• No alternate diagnosis that could better explain the neurological symptoms.^6^

The development and refinement of brain and spinal cord MRI throughout the 1980s and 1990s allowed for the detection of subclinical disease activity in patients who had experienced only a single clinical attack (clinically isolated syndrome, CIS). The first edition of the McDonald criteria, published in 2001, introduced the concept that in some patients with CIS, disease activity observed by MRI could suffice to establish a diagnosis of MS.^7^

The McDonald criteria have since undergone two revisions, the most recent in 2010, with the goal of incorporating the most up-to-date clinical knowledge and making the criteria simpler to use while maintaining their sensitivity and specificity.^8^ ^-^ ^10^ Table 1 summarizes the 2010 McDonald criteria and their definitions of DIS and DIT. Briefly, DIS can be fulfilled if MRI shows lesions in two of four characteristic areas (periventricular, juxtacortical, infratentorial, and spinal cord). MRI findings meet DIT criteria if a new lesion (T2 or gadolinium-enhancing) is observed on a follow-up scan, or if a single gadolinium-enhanced scan contains both enhancing (new, active) and nonenhancing (older, inactive) lesions, indicating at least two demyelinating events.^9^

**Table 1:** Summary of 2010 McDonald criteria

Clinical presentation	Additional MRI data needed for MS diagnosis
Two or more attacks; objective clinical evidence of two or more lesions or objective clinical evidence of one lesion with reasonable historical evidence of a prior attack	None, but strongly recommended to have a brain MRI with supportive features
Two or more attacks; objective clinical evidence of one lesion	**Dissemination in space** (DIS) demonstrated by one or more lesions in two of four characteristic areas (periventricular, juxtacortical, infratentorial, or spinal cord)
One attack (clinically isolated syndrome, CIS); objective clinical evidence of two or more lesions	**Dissemination in time** (DIT) demonstrated by:∙Simultaneous presence of enhancing and nonenhancing lesions OR∙New T2 and/or gadolinium-enhancing lesion at follow-up
One attack (CIS); objective clinical evidence of one lesion	DIS and DIT as described previously

Adapted from Polman et al, 2011.^9^

## MRI and Disease Management

Compared with earlier versions of the McDonald criteria that required a follow-up MRI to demonstrate DIT, the 2010 revision enables earlier diagnosis of MS in 30% to 50% of cases with classic CIS and a single contrast-enhanced MRI study. This has important implications not only for management decisions for individual patients, but also for larger issues such as the interpretation of clinical trial results.^11^ Early diagnosis and treatment have been linked to improved patient outcomes; trials of available disease-modifying therapies have shown that early treatment initiation is associated with delayed conversion from CIS to clinically definite MS^12^ ^-^ ^16^ and in the longer term with slower disease progression on the Expanded Disability Status Scale.^17^

The possibility of diagnosing MS earlier does not necessarily mean it must be treated earlier, but in some cases earlier diagnosis will lead to earlier treatment. This decision should still take into account additional factors including the overall clinical picture, diagnostic certainty, patient-related factors (e.g. attitudes toward treatment, likelihood of treatment adherence), and reimbursement conditions.^11^

The diagnostic criteria have their greatest validity and reliability when applied to patients younger than age 50 with a typical clinical syndrome consistent with demyelination of the central nervous system, such as optic neuritis, transverse myelitis, and brainstem syndromes (e.g. internuclear ophthalmoplegia) and “no better explanation” for the clinical condition other than MS. The supportive imaging criteria should be applied with caution in clinically ambiguous syndromes that may or may not ultimately evolve into definite MS.

In addition to its role in diagnosis, MRI can also monitor subclinical disease activity in CIS and MS and provide important information for ongoing patient management. However, there has historically been great variation among centres with regard to how and when MRI is used. This variability has made it difficult to compare findings across different centres, or even within the same centre if protocols or equipment change over time. Likewise, variations in imaging procedures over an individual patient’s disease course can complicate the interpretation of scans, particularly when searching for evidence of DIT.

To address the challenge of variability in the use of MRI in MS diagnosis and monitoring, an expert panel of the Consortium of Multiple Sclerosis Centres (CMSC) created a standardized MRI protocol that was first published in 2003^18^ and has since undergone two updates in response to advances in MRI technology and techniques.^19^ ^,^ ^20^ The CMSC is a network of more than 200 centres and 4000 health care professionals providing MS care to more than 150,000 patients in North America and Europe.

## The CAN-MRI-MS Consensus Panel

Although the 2010 McDonald criteria and CMSC standardized protocol have been available for several years, surveys of clinical practice in Canada and elsewhere^21^ show that their adoption has not always been optimal. To address this situation, a Canadian expert panel (CAN-MRI-MS Panel) was established with the goal of providing general practitioners and specialists (neurologists and radiologists) with expert consensus recommendations that clarify how and when MRI can be effectively used in the diagnosis and management of MS and CIS.

This article focusses on how the McDonald criteria and CMSC protocol can be effectively used in the Canadian clinical context and how communication and collaboration between neurologists and radiologists can be improved to enhance patient care for individuals with CIS or MS. Our recommendations are consistent with those developed by similar expert panels in Europe,^22^ ^,^ ^23^ but are tailored to Canadian clinical practice.

## Methods

The consensus statements presented in this article were developed by the CAN-MRI-MS Panel, an expert group of Canadian neurologists and radiologists who met in Vancouver, British Columbia, in September 2012. The meeting was designed to address one overarching question, namely, “How can neurologists and radiologists best use MRI for the diagnosis and management of MS patients in Canada?”

To further explore this central question, the Panel participated in a series of workshops and focused discussions aimed at collecting expert guidance and clinical best practices regarding the following objectives:

1. To review the McDonald 2010 criteria and make recommendations so that they will be useful, useable, and used.
2. To review the CMSC-standardized MRI protocol and make recommendations so that it will be useful, useable, and used.
3. To discuss and make recommendations about how to improve communication between neurologists and radiologists.

## Consensus Statements of the CAN-MRI-MS Panel

Eight brief consensus statements are presented (summarized in Table 2), each followed by points of clarification or disagreement when there was no unanimous consensus.

**Table 2:** Consensus statements of the CAN-MRI-MS panel regarding MRI use in MS diagnosis and management

Consensus statement	
**#1**	The CAN-MRI-MS Panel unanimously endorses the use of the McDonald 2010 criteria for the diagnosis of MS.
**#2**	The subcallosal plane is essential for the prescription of all axial sequences and a standardized core MRI sequence that allows for comparison of studies over time and across centres.
**#3**	Gadolinium imaging is useful for diagnosis of CIS and management of MS.
**#4**	The appropriate wait time for a diagnostic brain MRI for patients with new typical CIS is 1 week. In some clinical settings an MRI scan is required immediately.
**#5**	Patients with established relapsing–remitting MS should have, at minimum, a follow-up brain MRI:∙At 6 to 12 months after treatment switch ∙Annually while on disease-modifying treatment∙When there is unexpected clinical deterioration.
**#6**	The referring physician should include on the brain MRI requisition the following key clinical information:∙Purpose of the scan (diagnosis or follow-up of MS)∙Clinical scenario (definite CIS [syndrome and probable location] and date of CIS)∙If not classic CIS/MS, then clinical suspicion (likely or unlikely MS) and duration of symptoms∙Need for gadolinium and reason for request
**#7**	The radiology report should include:∙Descriptive elements (e.g. number and location of lesions, enhancing lesions, new lesions)∙Interpretation that can be used to support the clinical picture
**#8**	The CAN-MRI-MS Panel unanimously recommends that:∙Copies of brain MRI studies be retained permanently and be available to the patient’s health care team∙Patients be encouraged to keep their own scans on portable digital media

CIS: clinically isolated syndrome; MS: multiple sclerosis.

### *Consensus Statement #1: The CAN-MRI-MS Panel unanimously endorses the use of the McDonald 2010 criteria for the diagnosis of MS*

The 2010 McDonald criteria represent the most up-to-date and clinically relevant guidelines for using MRI to support a diagnosis of MS in patients with a single clinical attack suggestive of MS (CIS). Our Panel agrees that, in the appropriate clinical setting, the updated McDonald criteria will be simpler to use than previous versions and will be helpful for all physicians who manage patients with CIS/MS. In some cases, the 2010 McDonald criteria will enable earlier diagnosis of MS, potentially with a single brain MRI, if both DIS and DIT criteria are met.^9^

### The McDonald Criteria in Canadian Practice

The Panel noted that there are certain caveats for applying the criteria as well as areas in which the guidance should be adapted to fit within the reality of Canadian clinical practice. In particular, the McDonald criteria are applicable only in a specific clinical setting: evaluating patients with a typical CIS (i.e. subacute onset of optic neuritis, transverse myelitis, or brainstem syndrome). If the criteria are applied in the wrong population, for example the “query MS” cohort (i.e. patients with chronic or intermittent neurologic symptoms that may or may not be classic for MS), there is a risk of misdiagnosis because MRI is extremely sensitive for detecting any abnormality but lacks pathologic specificity.

With the development of more powerful equipment and more sensitive sequences, it is now possible to detect very small lesions that may not be clinically relevant. The original (2001) McDonald criteria state that “lesions will ordinarily be larger than 3 mm in cross section”;^7^ our Panel agrees with this definition and wishes to emphasize that lesions should be counted only if they are 3 mm or larger.

One area in which the recommendations and assumptions in the McDonald criteria may not fully reflect real-life practice is the use of gadolinium-containing contrast-enhancing agents. In current clinical practice, gadolinium-enhanced scans are not part of the routine diagnostic workup in many centres. Indeed, in many parts of Canada, access to gadolinium is limited and must be prioritized based on clinical need. Therefore, it will not always be possible to apply the elements of the DIT definition that involve gadolinium enhancement. We consider the use of gadolinium recommended but not essential in establishing the diagnosis of MS, as discussed further in Consensus Statement #3. One potential strategy to optimize resource use while minimizing diagnostic delays is to perform unenhanced examinations as a first-line triaging tool and reimage later with gadolinium if deemed necessary according to the unenhanced MRI findings.

Access to MRI equipment and MS experts is another factor that may limit the timely application of the McDonald criteria in Canada. Because of limited resources and geographic challenges, many patients wait longer for diagnosis than is optimal. Consensus Statement #4 recommends that a diagnostic MRI for CIS be prioritized to be done within one week of symptoms; Consensus Statement #5 summarizes our guidance on appropriate wait times and scheduling for follow-up scans.

### Communication Across Disciplines

Another key area not addressed by the McDonald criteria is continuity of knowledge among members of the health care team. The criteria and the clinical data that underlie them were developed by MRI experts with a particular interest in MS and incorporate the most advanced knowledge in the field. However, in real-life practice, it is unlikely that all members of the health care team will be equally conversant with these specialized concepts. It is therefore important to develop a common language and ensure that key concepts are generally understood, so that general practitioners, neurologists, radiologists, and other practitioners who care for CIS/MS patients can effectively communicate their needs and findings. Notably, it is crucial for all physicians applying these criteria to know how to define and identify CIS in order to use the correct terminology on reports and ensure that the criteria are being applied in an appropriate population. Consensus Statements #6 and #7 provide more detail about how to enhance communication between the clinicians requisitioning MRI scans and the radiologists reporting the results.

With their focus on the MRI-based definitions of DIS and DIT, the McDonald criteria often underplay the importance of other imaging results that also have clinical value. Our Panel encourages neurologists and radiologists to evaluate not only the lesion parameters that inform the DIS and DIT definitions, but also standard attributes such as size and morphological features that are not emphasized in the McDonald criteria. Clinicians requesting scans should also have access to all relevant images, not only the report, and should be encouraged to review them to have the most complete information available to support the clinical diagnosis. Important differential diagnoses that may mimic MS on imaging are reviewed elsewhere.^24^

Additionally, the McDonald criteria offer no guidance on interpreting MRI findings in patients with nonspecific white matter changes resembling MS lesions. Our Panel recommends that physicians be particularly careful to apply the appropriate clinical evaluation in these cases to avoid attributing nonspecific changes to MS-related pathology. Although MS-related lesions can vary in location and appearance, clinicians should recall the most typical presentation: an ovoid shape at least 3 mm across, most characteristically located in the periventricular, corpus callosum, brainstem, and/or juxtacortical regions. A fuller review of the features of MS lesions is available elsewhere.^4^ Other diseases may have a similar appearance, while in the earlier stages of MS the MRI findings may be minimal. Nonspecific white matter lesions, or unidentified bright objects, tend to be small, punctate, and randomly located throughout the white matter. They are present in 5% of the general population and increase in prevalence with age. These overlapping MRI features of lesions highlight the importance of exercising caution when interpreting MRI findings in the absence of a thorough clinical evaluation. For this reason, we have recommended that the radiology report generally avoid terms such as “MRI is diagnostic of MS” unless there is sufficient and specific clinical information provided to come to that conclusion (Consensus Statement #7).

#### Consensus Statement #2: The subcallosal plane is essential for the prescription of all axial sequences; a standardized core MRI sequence will allow for comparison of studies over time and across centres.

**Figure 1:**
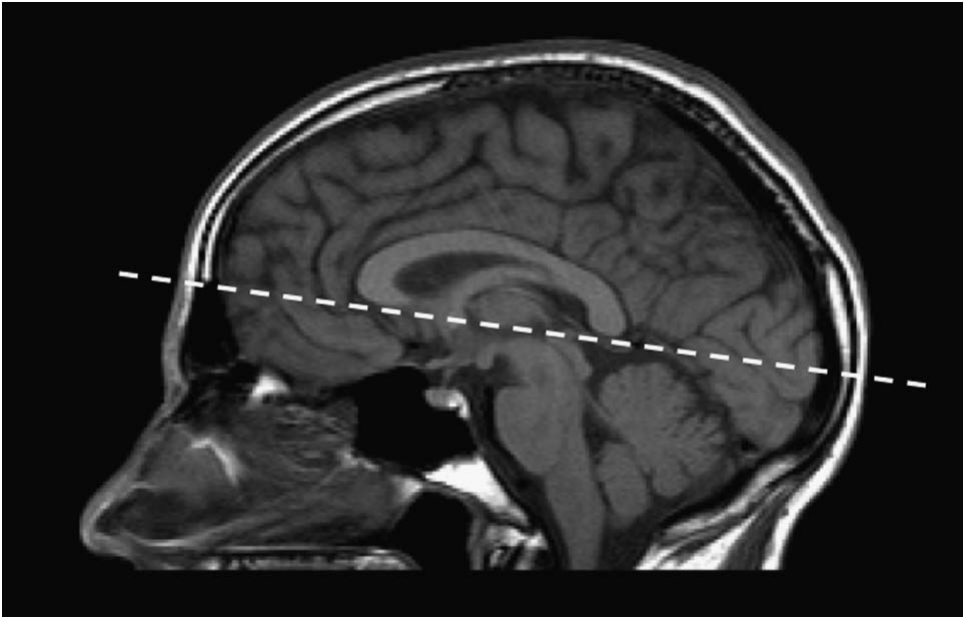
Sagittal midline localizer scan showing the subcallosal plane.

A standardized MRI protocol is essential for reliably detecting new clinically silent disease activity as part of the routine follow-up of MS patients. One of the most common issues in MS imaging follow-up is the difficulty of comparing examinations with different technical parameters because a significant proportion of lesions are only few millimetres in size and may not be revealed if the spatial resolution and the acquisition plane are not optimized. The most critical element of the MRI protocol is to consistently use a standard plane of orientation for all studies, namely the subcallosal plane (Figure 1) as recommended by the CMSC standardized MRI protocol (Table 3). Prescribing all axial sequences along the subcallosal plane will improve the reproducibility and comparability of scans, even when there are differences between studies with regard to equipment and/or slice thickness. The core sequence should be an axial FLAIR sequence oriented along the subcallosal plane with 3-mm contiguous slices (acquired directly from a two-dimensional sequence or from isotropic three-dimensional series); 4- to 5-mm slices with a maximum 1-mm gap, although less optimal, are also acceptable to satisfy limited scanning time requirement. Additional sequences including sagittal (FLAIR, proton-density–weighted, or T2), and another axial sequence (proton-density–weighted or T2) can be performed according to the CMSC guidelines or as specified by each centre’s protocols. There is no specific recommendation for field strength, as long as the scans are of good quality with adequate signal-to-noise ratio and spatial resolution (in-plane resolution should be ≤1 mm×1 mm).^20^

**Table 3:** Highlights of the CMSC-standardized protocol for brain MRI in CIS/MS

**Core principle**	∙Axial sequences oriented along the subcallosal plane (Figure 1)∙Whole brain coverage
**Core sequence**	∙Axial FLAIR:∘3-mm contiguous slices, acquired directly from a two-dimensional acquisition or from isotropic three-dimensional series∘4- to 5-mm slices with a maximum of 1-mm gap are also acceptable (although less optimal)
**Additional sequences**	∙Can be performed according to the CMSC guidelines or be specific to individual centres∙May include:∘Sagittal (FLAIR, PD, or T2)∘Axial T2∘Axial T1 (pre- and postcontrast)∙Optional:∘3D T1∘DWI
**Gadolinium enhancement**	∙Useful in certain clinical situations, but not always required (see Consensus Statement #3)
**Field strength**	∙Scans must be of good quality, with adequate SNR and spatial resolution (in plane 1 mm×1 mm)

CIS: clinically isolated syndrome; CMSC: Consortium of Multiple Sclerosis Centres; DWI: diffusion-weighted imaging; FLAIR: fluid-attenuated inversion recovery; PD: proton-density–weighted; SNR: signal-to-noise ratio.

Additionally, the CMSC protocol recommends that T1-weighted imaging with gadolinium enhancement be performed to assess DIT in patients with suspected MS and to monitor ongoing disease activity in patients with established MS.^20^ However, this recommendation diverges from current Canadian clinical practice: gadolinium enhancement is not part of the routine protocol in most centres. We offer our recommendations on the use of gadolinium in Consensus Statement #3.

A suggested ordering of sequences would include three-dimensional T1 precontrast, diffusion-weighted imaging (if needed, with recommended precontrast for progressive multifocal leukoencephalopathy surveillance), axial T2, gadolinium contrast injection, three-dimensional FLAIR (or axial and sagittal two-dimensional FLAIR), and postcontrast T1 (minimum five-minute delay after injection). Three-dimensional T1 is acquired in anticipation of future automatic volumetric analysis for brain atrophy. Analyzing this sequence after contrast is not compatible with many quantitative brain volume tools.

#### Consensus Statement #3: Gadolinium imaging is useful for diagnosis of CIS and management of MS.

Contrast enhancement with gadolinium provides information not available in other types of scans. It provides evidence of recent inflammatory activity that could be missed by relying on FLAIR sequence, particularly when nonstandardized studies are compared or if no prior imaging is available. The Panel’s preference is to use gadolinium whenever possible and clinically appropriate. However, current limited access and the expense of additional scans may make it necessary to prioritize gadolinium-enhanced scans based on patients’ clinical profiles and treatment-related factors. Table 4 summarizes the clinical situations in which the Panel deemed gadolinium either essential or recommended (but non-essential) in the diagnosis and monitoring of CIS and MS.

**Table 4:** Consensus guidance on appropriate use of gadolinium in patients with CIS and MS

Use of gadolinium	Clinical situations	
	CIS	MS
Gadolinium enhancement is **essential**	∙Atypical clinical presentation or unusual brain MRI features	∙Pretreatment for high-risk drug (e.g. mitoxantrone)∙Monitoring safety and/or failure on high-risk drug∙When required to meet treatment eligibility criteria∙When knowledge about recent ongoing disease activity is needed for management∙If unable to compare serial scans for new T2 lesions because of technical differences
Gadolinium enhancement is **recommended**	Provided that there are two or more white matter lesions present on brain imaging, enhancement should be considered:∙When there is potential for early diagnosis on a single scan∙When the results might affect treatment decisions∙When the results might affect patient counselling	∙Detecting new clinically silent disease activity in RRMS patients on DMT or considering starting DMT∙Establishing new baseline in natalizumab patients switching to another DMT (see Statement #5)

CIS: clinically isolated syndrome; DMT: disease-modifying therapy; MS: multiple sclerosis; RRMS: relapsing–remitting multiple sclerosis.

In patients with CIS, the consensus is that gadolinium enhancement is essential in individuals whose clinical presentation is atypical or in whom other brain imaging shows unusual features. In these cases, the presence or absence of enhancing lesions will be important information for ruling out other possible causes of the neurological symptoms, because many of the possible alternate diagnoses do not involve significant inflammation in the central nervous system.^4^ ^,^ ^24^ One potentially helpful practice is to perform only unenhanced imaging at the first set of scans and then to recall patients within the following month for a further gadolinium-enhanced scan if required, based on the initial findings.

In CIS cases with two or more white matter lesions 3 mm in size or greater that meet DIS criteria, we recommend the use of gadolinium because it may provide information useful for early diagnosis, treatment, or counselling. The 2010 McDonald criteria allow for a diagnosis of MS after a single clinical attack if enhancing and nonenhancing lesions are simultaneously seen on a single scan and DIS criteria are also met;^9^ the use of gadolinium will therefore enable earlier diagnosis of MS in 30% to 50% of CIS patients. Given the observed link between patients’ burden of enhancing lesions and their short-term relapse risk,^25^ gadolinium may be helpful for guiding counselling and/or treatment decisions in patients with numerous enhancing lesions.

In patients with an established diagnosis of MS, we recommend that gadolinium enhancement be considered as essential for several aspects of disease management and monitoring. In patients receiving high-risk drugs such as mitoxantrone, enhanced scans should be conducted before treatment to provide a baseline evaluation of current disease activity and also during therapy to monitor safety, efficacy, and possible treatment failure.^26^ Gadolinium may also be required to confirm a patient’s eligibility for a particular treatment option, based on the drug’s indication and local reimbursement conditions. It should also be considered essential whenever knowledge of ongoing disease activity could affect management decisions or when a gadolinium-enhanced scan could provide key information that is otherwise missing (e.g. if the patient has had serial scans for new T2 lesions that cannot be compared because of technical differences). Finally, the Panel suggested that gadolinium enhancement is helpful but not essential for detecting new but clinically silent disease activity in patients with established RRMS, especially in the context of assessing therapeutic response.^27^ Given all the potential benefits of gadolinium-enhanced studies, it is essential to establish good communication between neurologists and radiologists to ensure the use of proper protocols that are tailored to patient-specific situations while optimizing the use of available resources.

#### Consensus Statement #4: The appropriate wait time for a diagnostic brain MRI for patients with new typical CIS is one week. In some clinical settings an MRI scan is required immediately.

The Panel’s recommendation is that patients with a new typical CIS should receive a diagnostic brain and/or spinal cord MRI within a week of their first presentation. Clinicians should also be aware that in some clinical situations a one-week delay may not be acceptable and imaging should be performed immediately (e.g. when it is necessary to rule out spinal cord compression).

If the clinical presentation is atypical with nonlocalizable symptoms or signs, the appropriate wait time is less clearly defined and should be at the physician’s discretion, based on duration and severity of symptoms. For example, in a “query MS” patient with chronic or suspicious but clinically less characteristic MS symptoms, it may be appropriate to consider the priority the same as that of any other routine elective brain MRI. However, if there is a clinical basis for a stronger suspicion of MS, a wait time between one week and the wait for a routine scan would be applicable.

The use of high-dose corticosteroids can affect the MRI appearance for four to six weeks; this consideration should be kept in mind if steroids are used in response to the first clinical presentation. In some cases, it may be more appropriate to ensure the MRI scan is completed prior to initiation of steroids.

#### Consensus Statement #5: Patients with established RRMS should have, at minimum, a follow-up brain MRI:

∙ At 6 to 12 months after treatment switch
∙ Annually while on disease-modifying treatment
∙ When there is unexpected clinical deterioration

In patients with an established diagnosis of RRMS, the Panel’s consensus was that it is not necessary to schedule MRI investigation of every relapse. However, the Panel agreed that MRI can play a key role in monitoring clinically silent disease activity, particularly any changes that may occur in response to treatment switches. At the time of switching therapies, it can take anywhere from one to six months before the treatment is fully effective. New MRI activity occurring during that first six-month window may not best reflect the effectiveness of that therapeutic choice. We recommend that an MRI be obtained, with gadolinium enhancement if possible, approximately 6 to 12 months after a treatment switch to establish a new baseline against which any new lesion activity can be compared. (Depending on available resources and the patient’s clinical situation, it may be worth considering obtaining the new baseline sooner, approximately three to six months after switching.) If the scan shows gadolinium-enhancing lesions or new T2 lesions compared with a prior MRI, the MRI should be repeated four to six months later. While on treatment, an MRI should be performed annually for several years, and less frequently thereafter for clinically stable patients (e.g. every two to three years).

MRI can also play an important role in investigating deterioration because of very active inflammatory MS or any unexpected disease progression or breakthrough activity that cannot be adequately explained by MS: for example, investigating for complications of therapy such as progressive multifocal leukoencephalopathy or the development of a secondary condition such as a brain tumour.

In the case of patients previously treated with natalizumab who are discontinuing it and switching to a new disease-modifying therapy, a follow-up scan with gadolinium enhancement may be valuable to look for any changes suggestive of progressive multifocal leukoencephalopathy and establish a new “post-natalizumab baseline”.

#### Consensus Statement #6: The referring physician should include on the brain MRI requisition the following key clinical information:

∙ Purpose of the scan (diagnosis or follow-up of MS)
∙ Clinical scenario:
  ∘ Definite CIS (syndrome and probable location, date of CIS)
  ∘ If not classic CIS/MS, then clinical suspicion (likely or unlikely MS) and duration of symptoms
  ∘ RRMS follow-up (describe rationale for scan)
∙ Need for gadolinium and reason for request

The Panel further notes that requests to “rule out” or “query” MS are too vague to be useful unless accompanied by the information presented previously.

Standardized diagnostic criteria and MRI protocols can only succeed if communication among treating physicians is effective, particularly between the physicians who refer patients for MRI and the radiologists who report the results. The Panel’s radiologist members provided recommendations for neurologists about what types of information they prefer to receive—or not to receive—on an MRI requisition form. In general, our radiologist members felt it was useful to be given details of the overall clinical scenario, as outlined in Table 5. In particular, radiologists wanted a clear recommendation from referring physicians about whether a gadolinium-enhanced scan was required and the clinical rationale for such a request. The panellists found that requests to “rule out MS” or “query MS” were generally not helpful unless further clinical details were made available.

**Table 5:** Radiologists’ recommendations for an effective MRI requisition

What to write	What *not* to write
∙Purpose of the scan: Diagnosis or follow-up	∙“Query MS” or “rule out MS” without further clinical context
∙Clinical scenario:∘Diagnostic:▪For definite CIS, describe the syndrome (e.g. optic neuritis, transverse myelitis) and probable location, date of onset▪If not classic, then describe clinical suspicion (likely or unlikely MS) and duration of symptoms∘RRMS follow-up:▪State reason for follow-up (e.g. treatment monitoring, active inflammatory disease or unexpected deterioration, PML surveillance)▪May be useful to outline patient’s treatment history if purpose of MRI is to assess treatment response	
∙Whether or not gadolinium is required, and, if so, the clinical reason	∙Request for gadolinium without clinical rationale
∙Any special or optional sequences requested and the clinical rationale	∙Standard sequences—already specified by CMSC or local protocol
∙Whether or not spinal cord imaging is required	
∙Date, location, and any relevant results from previous imaging	

CIS: clinically isolated syndrome; CMSC: Consortium of Multiple Sclerosis Centres; MS: multiple sclerosis; PML: progressive multifocal leukoencephalopathy.

#### Consensus Statement #7: The radiology report should include:

∙ Descriptive elements (e.g. number and location of lesions, enhancing lesions, new lesions)
∙ Interpretation that can be used to support the clinical picture

As a complement to Statement #6 regarding requisitions, the neurologist members of the Panel provided insight into the types of information and interpretation that they found useful on MRI reports from radiologists (Table 6). In general, neurologists wanted to see objective descriptions of key findings including lesion number and location as well as the presence and location of any new and/or enhancing lesions. The neurologist members of the Panel also welcomed interpretation of the results that could be used to complement the clinical evidence (e.g. “lesions suggestive of MS”, “lesions typical of MS”). However, they cautioned against more definitive diagnostic statements (e.g. “lesions diagnostic of MS”) because diagnosis must also take into account clinical information that may not have been available to the radiologist. Similarly, unless the requisitioning physician specifically asked for interrogation of the McDonald criteria for DIS or DIT, reports should avoid wording such as “McDonald criteria for diagnosis of MS met” because the criteria are relevant only when applied in a clinically appropriate population.

**Table 6:** Neurologists’ recommendations for an effective MRI report

What to include	What *not* to include
∙Descriptive elements:∘Number of lesions (e.g. 0-10, >10, numerous)∘Location (e.g. juxtacortical, subcortical/deep, periventricular, infratentorial)∘Comment on whether lesions are typical of demyelination∘Number and location of enhancing lesions∘Number and location of definite new (≥3 mm) and/or enlarging lesions compared to previous study∘Pattern of lesions∘Presence and appearance of any spinal cord lesions∘Comment on whether scan meets DIS or DIT criteria (if requested)	∙Focus on irrelevant or incidental findings∙Vague descriptions (e.g. “changes consistent with demyelination”) without further details
∙Interpretation:∘Preferred wording is “lesions typical of” or “suggestive of” MS∘Commentary welcomed on whether lesion pattern is suggestive of active disease∘Useful to compare/contrast with earlier scans if available∘Should report any findings that are **not** typical of MS, to aid in differential diagnosis	∙“Lesions diagnostic of MS”∙“McDonald MS criteria met”
∙Reinforcement of the need for clinical information to support MRI findings in diagnosis	∙A diagnosis based on MRI findings alone

DIS: dissemination in space; DIT: dissemination in time; MS: multiple sclerosis.

#### Consensus Statement #8: The CAN-MRI-MS Panel unanimously recommends that:

∙ Copies of brain MRI studies be retained permanently and be available to the patient’s health care team
∙ Patients be encouraged to keep their own scans on portable digital media

As with any chronic health condition that must be managed over the long term, continuity of care plays a crucial role in the diagnosis and treatment of patients with CIS and MS. With the increasing importance of MRI findings in diagnosing, monitoring, and managing these conditions, we recommend that all brain MRI studies be documented and these records retained permanently with the patient’s file. MRI records should be made readily accessible to all relevant members of the healthcare team. Additionally, it may be beneficial for patients to keep a copy of their own results on portable digital media (e.g. CD-ROM, USB drive), both for their own information and to help ensure continuity if they receive care from health professionals outside their usual team.

## Discussion

With the growing and evolving role of MRI in the diagnosis and monitoring of patients with CIS and MS, it will become increasingly important for health care professionals who manage these patients to communicate effectively and make use of the available clinical evidence and best practices. We therefore call on neurologists and radiologists in Canada to incorporate the McDonald criteria, the CMSC MRI protocol, and other guidance given in this consensus paper into their practices. For these recommendations to translate into improved patient care, it is important that they be applied not only by the specialists who provide the majority of ongoing care for patients with CIS and MS, but also by the general practitioners who encounter CIS/MS patients in their daily practice and are often involved in the initial diagnosis and workup (including the first MRI requisition) of these conditions.

Our hope is that by improving communication and general awareness of best practices for MRI use in MS diagnosis and monitoring, we can improve patient care across Canada by providing timely diagnosis, informed management decisions, and better continuity of care.
